# Positive effects of hydrogen-water bathing in patients of psoriasis and parapsoriasis en plaques

**DOI:** 10.1038/s41598-018-26388-3

**Published:** 2018-05-23

**Authors:** Qinyuan Zhu, Yueshen Wu, Yongmei Li, Zihua Chen, Lanting Wang, Hao Xiong, Erhong Dai, Jianhua Wu, Bin Fan, Li Ping, Xiaoqun Luo

**Affiliations:** 1 Department of Dermatology, Huashan Hospital, Fudan University, 12 Middle Wulumuqi Rd., Jing’an District, Shanghai, China; 2 Department of Dermatology, Huadong Hospital, Fudan University, 221 West Yan-an Rd., Jing’an District, Shanghai, China; 3 Department of Dermatology, Longhua Hospital, Shanghai University of Traditional Chinese Medicine, 725 South Wanping Road, Xuhui District, Shanghai, China; 4 Department of Dermatology, Shanghai Dermatology Hospital, 200 Wuyi Road, Chang-ning District, Shanghai, China; 5 Department of Dermatology, Changhai Hospital, The Second Military Medical University, 168 Changhai Rd., Shanghai, China; 6 Department of Dermatology, Yueyang Hospital of Integrated Traditional Chinese and Western Medicine, Shanghai University of Traditional Chinese Medicine, 110 Ganhe Road, Shanghai, China

## Abstract

Psoriasis and parapsoriasis en plaques are chronic inflammatory skin diseases, both representing therapeutic challenge in daily practice and adversely affecting the quality of life. Reactive oxygen species (ROS) has been evidenced to be involved in the pathogenesis of the chronic inflammatory diseases. We now report that hydrogen water, an effective ROS scavenger, has significant and rapid improvement in disease severity and quality of life for patients with psoriasis and parapsoriasis en plaques. At week 8, our parallel-controlled trial revealed 24.4% of patients (10/41) receiving hydrogen-water bathing achieved at least 75% improvement in Psoriasis Area Severity Index (PASI) score compared with 2.9% of patients (1/34) of the control group (*Pc* = 0.022, OR = 0.094, 95%CI = [0.011, 0.777]). Of patients, 56.1% (23/41) who received bathing achieved at least 50% improvement in PASI score compared with only 17.7%(6/34) of the control group (*P* = 0.001, OR = 0.168, 95%CI = [0.057, 0.492]). The significant improvement of pruritus was also observed (*P* = 3.94 × 10^−4^). Besides, complete response was observed in 33.3% of patients (2/6) of parapsoriasis en plaques and partial response in 66.7% (4/6) at week 8. Our findings suggested that hydrogen-water bathing therapy could fulfill the unmet need for these chronic inflammatory skin diseases.

## Introduction

Psoriasis and parapsoriasis en plaques are both chronic inflammatory skin diseases characterized by persistently scaling and inflammatory eruptions^1,2^. They represent therapeutic challenge in daily practice and adversely affect the quality of life of patients^3–6^. Psoriasis is so common that has been recognized since ancient times, affecting about 1% to 3% of the general population^7^. It is associated with a high degree of morbidity. Indeed, disability and impact on quality of life secondary to psoriasis parallels that of heart disease and arthritis^8,9^. Parapsoriasis en plaques is a relatively rare group of disorders which has been classified into small plaque parapsoriasis (SPP) and large plaque parapsoriasis (LPP) according to the size of the lesions. Although the relation of SPP with mycosis fungoides (MF) is still a matter of discussion, there are about 10–30% of cases of LPP result in MF finally^10–13^. The interactive network of the immune system and skin cells is thought to play a vital role in the pathogenesis of both diseases. To be more accurate, psoriasis is considered a Th1⁄Th17-driven disease^11–13^, while parapsoriasis en plaques is a model of cutaneous T cell lympho-proliferative disorders and has been proved to be a monoclonal disorder in many cases. For a long period, conventional treatment to both diseases has not fully met the needs of patients while having well-known side-effects. The improved understanding of the autoimmune inflammatory pathways and associated changing concepts in pathogenesis have led to the development of biological drugs, which especially revolutionized the treatment of psoriasis^4,14^. However, slow onset of action, high cost, efficacy lost over time and the long-term safety profile of these biologics still remain unsolved^3–5^.

Recently, it has been evidenced that oxidative stress such as increased reactive oxygen species (ROS) production may be involved in the pathogenesis of chronic inflammatory diseases^15,16^. The possibility of using this information to develop novel strategies for treatment is of considerable interest. Hydrogen molecule (H_2_) has been used in medical applications as a safe and effective antioxidant and immunomodulator with minimal side effects^16–18^. Unlike other antioxidants, which are unable to target organelles, H_2_ can penetrate biomembranes and diffuse into the cytosol, mitochondria and nucleus^19^. Moreover, it has also been reported to selectively scavenge ROS^17^ and show positive influence in Th1, Th2, and pro-inflammatory cytokine imbalance^20^. Up to date, hydrogen water (solubilized H_2_) as a treatment strategy for psoriasis-associated skin lesions has been tried by few case reports^21^, and neither has hydrogen water for patients with parapsoriasis en plaques. Apart from drinking hydrogen water, inhalation of hydrogen gas and injecting H_2_-dissolved saline, hydrogen-water bathing is a new approach highlights by its skin-directed, safe and painless administration. Thus, our study conducted a parallel-controlled trial in patients with psoriasis and a self-controlled trial in patients with parapsoriasis en plaques to evaluate the efficacy of hydrogen-water bathing to these chronic inflammatory skin diseases.

## Results

### Improvement of psoriasis

In all, 41 psoriasis patients were assigned to treatment with hydrogen-water bathing therapy and 34 patients were assigned to the control group. The treatment groups were well balanced with respect to demographics and baseline characteristics (Table 1). Only one patient of the control group withdrew during the course of the study at week 2 due to a lack of improvement and she was counted as a non-responder in the control group. Response was evident after 8-week bathing therapy. The mean Psoriasis Area Severity Index (PASI) score and median visual analog scale (VAS) score of the hydrogen-water bathing group at week 8 was 5.8 and 0 respectively, significantly lower than the baseline scores (*P* = 7.08 × 10^−6^; *P* = 2.42 × 10^−5^).

**Table 1:** Characteristics of the psoriasis patients.

	The Hydrogen-water bathing group		The control group	
	Baseline	Week 8	Baseline	Week 8
No	41	41	34	33
Sex(male/female)	24/17	24/17	18/16	18/15
Age	40 ± 15 (18–78)	40 ± 15 (18–78)	39 ± 12 (18–72)	39 ± 13 (18–72)
BMI	23.8 ± 3.8 (17.5–35.5)	23.7 ± 3.9 (17.2–35.6)	23.1 ± 4.2 (15.5–31.4)	23.0 ± 4.6 (15.3–31.4)
Waistline (cm)	82.7 ± 10.3 (63.3–103.3)	82.8 ± 9.8 (63.3–103.3)	76.8 ± 8.7 (58.2–95.4)	76.8 ± 8.9 (58.2–95.4)
PASI score	9.8 ± 5.9 (1.4–25.2)	5.8 ± 5.5 (0.2–25.2)	8.5 ± 4.1 (2.8–23.8)	7.9 ± 6.8 (0.8–34.5)
VAS score (median, range)	2 (0–8)	0 (0–4)	0 (0–7)	0 (0–9)

PASI: Psoriasis Area Severity Index; VAS: the visual analog scale; BMI: Body Mass Index.

**Figure 1:**
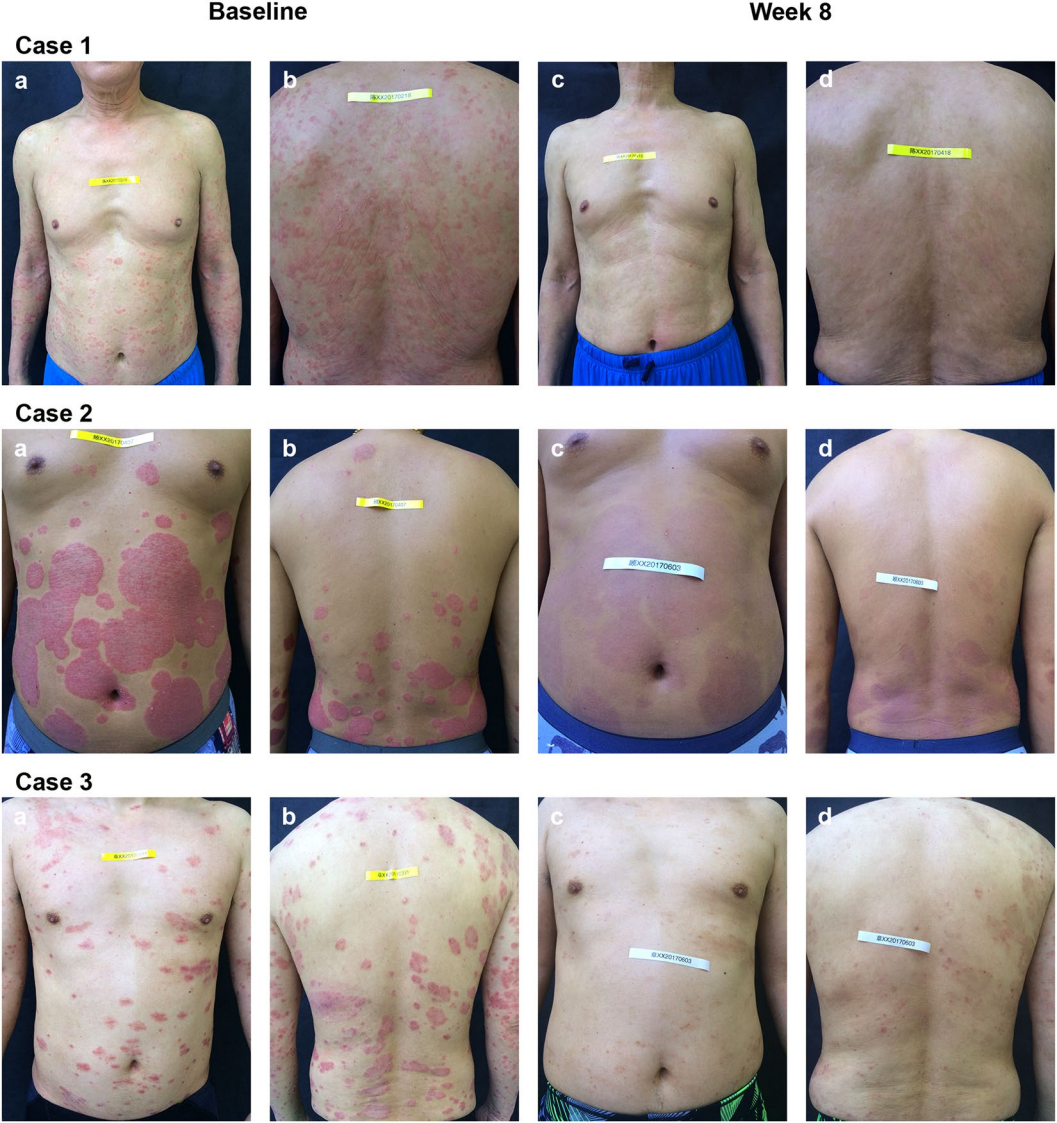
Clinical improvement of psoriasis of an 8-week course of hydrogen-water bathing therapy. **Case 1:** A 64-year-old psoriasis patient at baseline (PASI 16.4, **a**,**b**) and after the bathing therapy (PASI 1.8, **c**,**d**). Although he had been treated with acitretin capsules 30 mg daily for more than 4 months, the psoriatic lesions had not improved except for the partially reduced scale on the plaque. He refused to increase the drug dose due to intolerable dryness and chapping of the mucous membranes. **Case 2**: A 40-year-old psoriasis patient at baseline (PASI 21.1, **a,b**) and after the last bathing therapy (PASI 4.1, **c**,**d**). He complained of severely itching and treatment-resistant lesions (acitretin capsules 40 mg daily for more than 6 months), and after bathing therapy he was able to reduce the dose. **Case 3**: A 43-year-old psoriasis patient at baseline (PASI 20.2, **a**,**b**) and after the last bathing therapy (PASI 4.8, **c**,**d**). This man had been continuously treated with methotrexate 5 mg weekly for more than 10 months and was able to reduce the dose successfully after bathing therapy. Note that patients experienced similar responses in the areas not shown.

After 8 weeks of therapy, patients treated with hydrogen-water bathing showed significantly greater improvement than those who were of the control group as evaluated by both PASI and VAS (Table 2 and Fig. 1). Of patients, 24.4% receiving hydrogen-water bathing achieved the end point of at least 75% improvement in PASI score compared with 2.9% of patients of the control group (*Pc* = 0.022, OR = 0.094, 95%CI = [0.011, 0.777]). Of patients, 56.1% who received bathing achieved at least 50% improvement in PASI compared with only 17.7% of the control group (*P* = 0.001, OR = 0.168, 95%CI = [0.057, 0.492]). Hydrogen-water bathing treatment also resulted in substantial improvement in pruritus as measured by VAS. The median change from baseline to week 8 in the bathing group was −2, compared with a median change of 0 in the control group (*P* = 3.94 × 10^−4^).

**Table 2:** Summary of the improvement of Psoriasis Area and Severity Index (PASI) and visual analog scale (VAS) at week 8.

		The Hydrogen-water bathing group				The control group				*P* value
		Baseline PASI score				Baseline PASI score				
		Mild	Moderate	Severe	Total	Mild	Moderate	Severe	Total	
		(N = 26)	(N = 11)	(N = 4)	(N = 41)	(N = 24)	(N = 9)	(N = 1)	(N = 34)	
PASI (%)	>PASI90	1 (2.4)	1 (2.4)	0	2 (4.8)	0	0	0	0	>0.05
	>PASI75	5 (12.2)	3 (7.3)	2 (4.9)	10 (24.4)	1 (2.9)	0	0	1 (2.9)	0.022^*^
	>PASI50	13 (31.7)	8 (19.5)	2 (4.9)	23 (56.1)	4 (11.8)	2 (5.9)	0	6 (17.6)	0.001
VAS improvement (%)	≤−5	3 (7.3)				0 (0)				0.31^*^
	≤−3	9 (22.0)				1 (2.9)				0.04^*^
	<0	21 (51.2)				7 (20.6)				0.006
	≥0	20 (48.8)				27 (79.4)				0.006

^*^The corrected *P* (*P*c) values were adjusted by using Yate’s correction for continuity.

### Improvement of parapsoriasis en plaques

**Figure 2:**
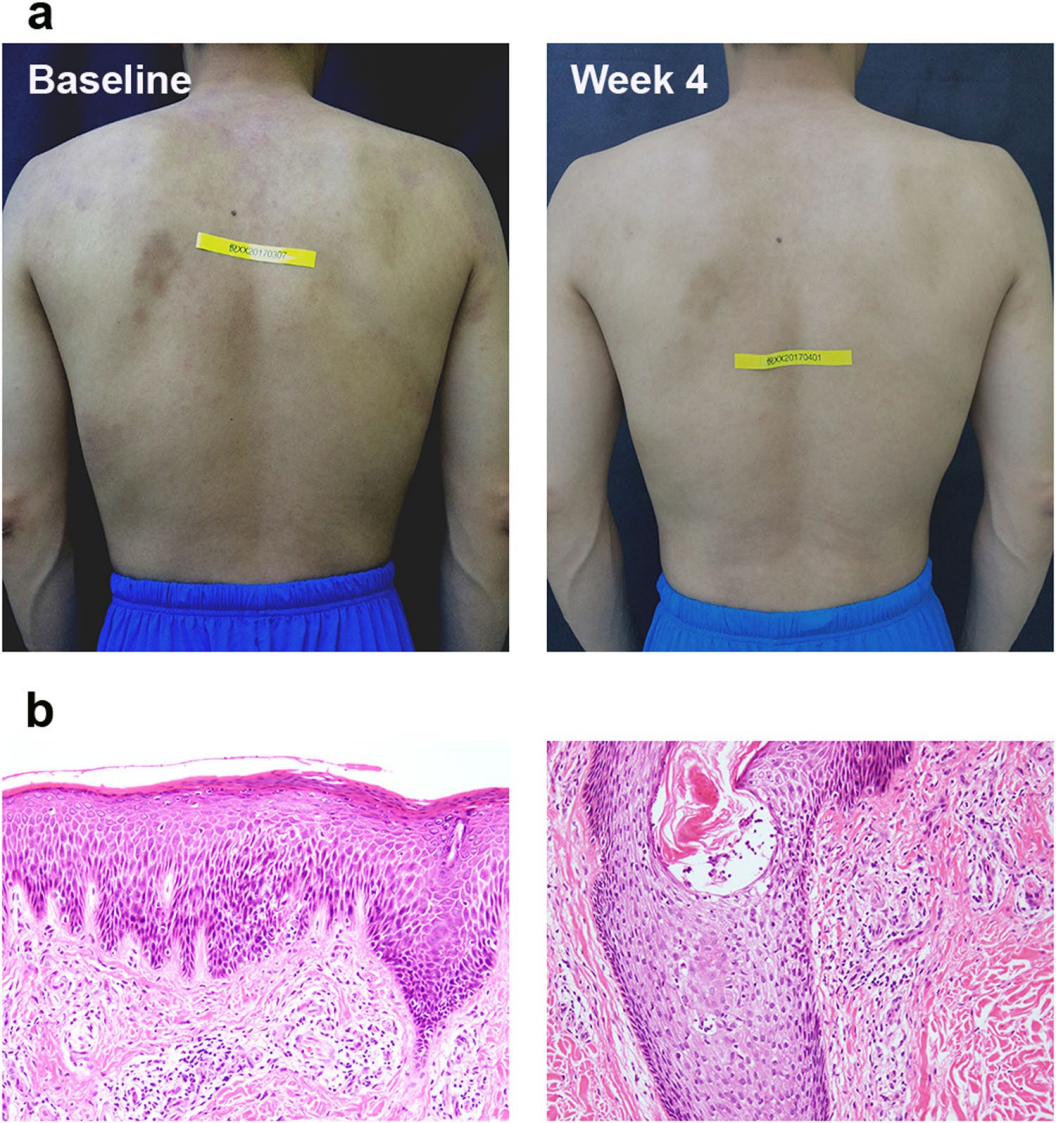
Clinical evaluation of a patient of parapsoriasis en plaques who achieved complete response rapidly 4 weeks after hydrogen-water bathing. A 35-year-old man with large plaque parapsoriasis had been followed up for 30 months and during that time two biopsies were taken showing no progression. He had suffered flare-up after 10-month narrow-band UVB therapy and failed to have evident improvement in the later 6-month phototherapy despite of increasing the power. Even if only 4 weeks, his lesions rapidly achieved significant improvements without concomitant therapy (**a**). The Hematoxylin-eosin stain shows mildly hyperkeratotic and focally parakeratotic epidermis with moderately dense superficial perivascular infiltrate. Lymphoid cells are mostly small, cytologically normal lymphocytes, and there is focal single-cell epidermotropism (**b**).

Six patients were included: 1 man and 5 women, with mean age of 32.8 ± 4.9 (range: 25–40) years and mean course duration of 34.4 ± 31.1 (range: 12–96) months. Four patients were categorized as LPP and two as SPP. Features of the patients were presented in Table 3. In all patients, an improvement in the morphology or distribution of lesions had occurred (Fig. 2). Complete response was observed in 33.3% of patients (2/6), partial response in 66.7% (4/6).

**Table 3:** Characteristics and the clinical outcomes of patients with parapsoriasis en plaques.

Patients	Sex/Age	Type of parasporiasis	Distribution at initial presentation	Morphology at initial presentation	Duration of disease (month)	Clinical response at week 8
1	F/40	LPP	trunk and extremities	patch, plaque	25	PR
2	F/31	LPP	trunk	papule, patch	12	PR
3	F/33	SPP	trunk and extremities	papule, patch, plaque	28	PR
4	F/33	SPP	trunk	papule, patch	15	PR
5	M/35	LPP	trunk and extremities	patch, plaque	30	CR
6	F/25	LPP	trunk and extremities	patch, plaque	96	CR

SPP: small plaque parapsoriasis; LPP: large plaque parapsoriasis; PR: partial response; CR: complete response.

### Adverse events

Two psoriasis patients complained of the temperature of the hydrogen water. The discomfort was relieved once the actual temperature was adjusted according to the satisfaction of patients. No other adverse reactions were found during the study.

## Discussion

The results of the parallel-controlled trial demonstrated that hydrogen-water bathing therapy led to significant improvements in psoriasis for the majority of patients. The response rate observed was obviously higher than those seen with Alefacept and fumaric acid esters; and was similar to those seen with Efalizumab, low dose of oral methotrexate (MTX) (5–15 mg/week) and cyclosporine A (1.25 mg/kg/day)^22–26^. Furthermore, patients receiving hydrogen-water bathing showed rapid onset of improvement from baseline. Approximately one forth of patients showed at least 75% improvement in PASI score 8 weeks after their initial bath, a level of response that has only been observed after 12 or more weeks of therapy in patients receiving some biologic agents^23,24,27^. Patients treated with hydrogen-water bathing also showed substantial improvement in pruritus as assessed by VAS. This is beneficial to the quality of life of psoriasis, which is considered to be similar to, if not worse than, that of other major chronic diseases. Although concomitant treatment was used for the skin lesions, it should be noted that the dosage of MTX, UVB phototherapy and systemic retinoids concomitantly used were not effective for at least 4 months prior to participation in the present study. Surprisingly, 6 patients were able to reduce or even stop the drug dosage (4 patients: acitretin; 2 patients: MTX) after the bathing course. Although the possibility that the improvements were caused by concomitant treatment cannot be fully excluded, it is indicated that the quick relief of symptom was in great part owing to the bathing therapy.

To parapsoriasis en plaques, our result suggested that hydrogen-water bathing was rapidly effective and safe for the control of the disease with 66.7% partial response and 33.3% complete response. Currently, PUVA and narrow-band UVB are used as main treatment options for parapsoriasis en plaques with up to 80% complete remission rates and a median time to clearance of 2–6 months^6,28,29^. In general, UVB is preferred in patients with patches and thin plaques and PUVA photochemotherapy should be used for patients with thick plaques, with phototypes ≥III and unresponsive to UVB^6^. However, in addition to requirement of long time to induce the response and the maintenance, all these therapies are associated with potential risk of photocarcinogenesis and photoaging limits their long-term use.

Psoriasis and parapsoriasis en plaques are known as representative diseases that show the orchestrated mechanisms of chronic inflammation. The clinical effectiveness of hydrogen-water may partially be explained by H_2_ selectively scavenger ability against highly active oxidants, such as hydroxyl radical and peroxynitrite, and cytoprotective effects against oxidative stress^17^. Hydroxyl radical is known as a major trigger of the chain reaction of free radicals^30^, and the absence of the specific scavenger of this species spontaneously causes oxidative states in chronic inflammation^31,32^. Thus, H_2_ may have an advantage to suppress the chain reaction, which produces lipid peroxide and leads to the generation of oxidative stress markers, such as malondialdehyde (MDA)^32^ which has been proved to be in association with the exacerbation of psoriasis^33^. Another target of H_2_, peroxynitrite, which is generated from the reaction of nitric oxide with superoxide, activates *p*38 MAPK pathways which are related to production of inflammatory cytokines, such as TNF-α,IL-6, IL-8 and many others^20^, resulting in the development of plaque of psoriasis^34^. Subsequent studies indicate that the effect of H_2_ is mediated by Nrf2-Keap1 system^35,36^, a transcriptional factor known to be an activator of intrinsic protective mechanisms against oxidative stress, but the mechanisms remain to be solved. However, radical scavenging effects of H_2_ cannot fully explain the anti-inflammatory and anti-apoptotic effects, which should involve a number of fine-tuned signaling pathways. Studies also have shown that H_2_ suppresses signaling pathways in allergies^37^ and inflammation^38^ without directly scavenging reactive oxygen/nitrogen species.

In fact, anti-oxidant therapies to psoriasis have already been tested, e.g. using fumaric acid esters particularly in Germany^39^. However, most of them exhibited limited therapeutic success. Furthermore, recent studies suggested that some ROS act as signaling messengers to regulate a wide variety of physiological process^40,41^. In view of this background, an ideal antioxidant is expected to mitigate excess oxidative stress, but not disturb redox homeostasis. H_2_ has the capability to scavenge specifically potent ROS but does not react with those that have important physiological roles^17^. The safety of H_2_ is also established by its intrinsic production in the human body and inertness against biogenic components. It has been already used for the prevention of decompression sickness in deep divers^42^. The clinical practice of H_2_ in the treatment of chronic inflammatory disease was recently attempted in patients of rheumatoid arthritis (RA)^43^. Moreover, a latest case report suggested H_2_ could relieve psoriasis-associated skin lesions and arthritis^21^. Apart from other methods of application, hydrogen-water bathing is a new approach highlights by its skin-directed, safe and painless administration and can be carried out in daily life.

Regarding the present study, our results showed a decreased trend of BMI in psoriasis patients treated with bathing therapy without any lipid-lowering interventions. This result matches those of previous studies, which have demonstrated the clinical improvement in patients with psoriasis was associated with a reduction in the levels of lipid peroxidation and an increased serum antioxidant capacity^44^. In addition, it should be noted that the itching sensation was markedly reduced in most cases. The influence of hydrogen water on itching sensation suggests the presence of neurogenic inflammation associated with ROS in the psoriatic lesion and the possibility of a therapeutic approach similar to that for neurological inflammatory disorders^17^. Some limitations of this study need to be pointed out. As an open trial of limited sample size, this study may include selection bias although the baseline characteristics of the psoriasis groups including primary PASI and VAS scores showed well balanced. Attention should be paid that the patients receiving hydrogen-bathing therapy are those who have failed to conventional treatment for more than 4 months. This at least implied the disease activities of these “refractory” patients were in less stable condition. Secondly, this study did not involve a placebo control group owing to the ethics concern. However, all the ones of the control group had received tap-water bathing more than twice a week during this study. Thus, the control group of psoriasis was administered with the combination therapy of the conventional therapy and the placebo (tap-water) bathing.

In summary, patients with psoriasis and parapsoriasis en plaques who were treated with hydrogen-water bathing therapy achieved significant and rapid improvement in disease severity and quality of life. We suggested that hydrogen-water bathing therapy could fulfill the unmet need for an alternative therapeutic option for these patients. Further large randomized placebo-controlled trials are required to verify and extend these results. The mechanism and long-term efficacy of hydrogen-water in these diseases are also warranted.

## Methods

### Patients

Forty-one patients of psoriasis and six patients of parapsoriasis en plaques were enrolled from February 2016 to April 2017 from Huashan Hospital affiliated to Fudan University and Huadong Hospital affiliated to Fudan University. The control group of psoriasis included thirty-four patients recruited from the dermatology clinics of Huashan Hospital. The study was registered and approved by China Ethics Committee of Registering Clinical Trials (ChiCTR-ONC-17013055, 2017/10/20). All patients signed an informed consent form and agreed to publish identifying information or images. All methods were performed in accordance with the relevant guidelines and regulation.

Patients of psoriasis had a history of plaque psoriasis for a minimum of 12 months. Among them, 21 patients were resistant to topical corticosteroid and calcipotriol ointments; the rest of patients suffered conventional treatment failure or failed to reduce the existing dosage of drugs beyond topical corticosteroid and calcipotriol ointments for more than 4 months. The failed therapeutic options include UVB phototherapy (10/41), MTX (3/41), and systemic retinoids (7/41). All the patients declined treatment of other drugs (include biologics) due to financial issues and safety concern. Patients of parapsoriasis en plaques were diagnosed based on clinical, histopathological and immunohistochemical findings (SPP: 2/6, LPP: 4/6). They had been followed up for more than 8 months. Among them, 4 patients had received UVA or narrow-band UVB therapy for more than 6 months without evident improvements. Two patients suffered flare-ups during phototherapy. All biopsies reported dense lymphocytic infiltrates, occasionally with lymphocyte exocytosis. None of the patients had axillary or inguinal lymphadenopathy. The laboratory results of all patients were unremarkable. Patients with serious cardiovascular diseases or infectious diseases, and those who were unable to receive treatment regularly were excluded.

During the duration of hydrogen-water bathing therapy, the present treatments of psoriasis patients were continued the same as before (except for drug tapering), including systemic and topical therapy. The patients of the psoriasis-controlled group were administered the same traditional Chinese patent medicine called “Qu-Yin oral solution”, topical treatment of corticosteroid and calcipotriol ointments. One major ingredient of this widely-used solution is glycyrrhizin, which has been evidenced to enhance the clinical response of psoriasis with its anti-inflammatory and immune-modulating effect^45^. All the ones of the control group had received tap-water bathing more than twice a week during this study. Patients of parapsoriasis en plaques did not use any concomitant therapy, except for topical corticosteroid and emollients.

### Hydrogen water bathing

Hydrogen-water bathing was administrated through skin by immersing whole body in the hydrogen-water twice a week (interval of 3 days). Each bathing took 10 to 15 minutes. Hydrogen bathing paused one week in case of menstruation in female subjects. The hydrogen-bathing machine (provided by Shanghai Yiquan Investment Limited Partnership) freshly prepared hydrogen water using nanobubble technology to dissolve hydrogen gas into pure deionized water. In briefly it contained the following process: (1) Tap-water passed through a filtration system (composed of quartz sand, activated carbon, ultrafiltration and reverse osmosis membrane) and an ultraviolet disinfection unit to be deionized and disinfected. (2) Hydrogen generator electrolyzed treated tap-water into oxygen and hydrogen and then collected pure hydrogen gas. (3) Hydrogen gas was forced into micro-nano-level bubbles and the bubbles were then dissolved directly and evenly into deionized water. The freshly prepared hydrogen water had the following physical and chemical characteristics: (1) pH 6.8–7.3. (2) Temperature ranged from 38 to 42 °C (the actual temperature based on the satisfaction of patients). (3) High content of dissolved hydrogen with a concentration of 1.0 ppm (for reference, the dissolved hydrogen of tap-water is less than 0.001 ppm). (4) With an extremely negative oxidation reduction potential (ORP) of −580 mV~ −650 mV (for reference, tap water: +250 mV~ +350 mV). Each time before therapy the same equipment was used to test pH, temperature, ORP (RM-30P, DKK-TOA Corp., Japan) and hydrogen concentration (ENH-1000, Trustlex Corp., Japan) to make sure hydrogen water having the same properties.

### Efficacy evaluation

#### Psoriasis

Clinical assessments including physical examinations, vital signs, concomitant medications, adverse events and measures of psoriasis activity (PASI scores and photos) were estimated at baseline and following each bathing treatment. For the PASI, patients are rated on the basis of erythema, scaling, and thickness divided in four anatomical parts (head, trunk, upper extremities, and lower extremities). The area of each anatomical part is factored into the overall value^46^. The score was divided into mild (1–10), moderate (10–20) and severe (>20) PASI. The PASI score at week 8 was the predefined efficacy endpoint, where a favorable response was an improvement of at least 50% from the baseline PASI^47^. The pruritus of the skin lesions was measured by the VAS for itching^48^.

### Parapsoriasis en plaques

Clinical responses were evaluated at week 8, classified as complete response, >90% clearance of lesions; partial response, 50–90% clearance; no response, <50% clearance with persistent skin lesions despite continuing treatment. The pruritus of the skin lesions was measured by VAS as well.

### Statistical analysis

Analyses of effectiveness endpoints were based on the intent-to-treat (ITT) population. The last-observation-carried-forward (LOCF) analysis was used to estimate the missing data for effectiveness variables. Descriptive variables were summarized by number (percentages), median or mean ± standard deviation. Measurement data were compared using paried t-test. Comparison of the count data or level data was performed using χ2 tests, Fisher’s exact tests or Mann-Whitney U tests. Odds ratio (OR) were calculated with Haldane’s modification, which adds 0.5 to all cells to accommodate possible zero counts^49^. *P* values were two-tailed. Differences were considered significant at *P* < 0.05. The corrected *P* (*P*c) values were adjusted by using Yate’s correction for continuity. Data were analyzed by SPSS17.0 (SPSS Inc., Chicago, IL, USA) software.

### Data availability

The datasets generated during and/or analyzed during the current study are available from the corresponding author on reasonable request.
